# *distect*: automatic sample-position tracking for X-ray experiments using computer vision algorithms

**DOI:** 10.1107/S1600577524009536

**Published:** 2024-10-30

**Authors:** Michael Berg, Dirk Furrer, Vincent Thominet, Xiaoqiang Wang, Stefan Zeugin, Helmut Grabner, Kurt Stockinger, Cinthia Piamonteze

**Affiliations:** a School of Engineering Zurich University of Applied Sciences Switzerland; b Paul Scherrer Institute 5232Villigen-PSI Switzerland; Advanced Photon Source, USA

**Keywords:** variable temperature inserts, sample-position tracking, soft X-ray absorption spectroscopy, computer vision algorithms, sample-position accuracy

## Abstract

Soft X-ray spectroscopy is an important technique for measuring the fundamental properties of materials. However, for measurements of samples in the sub-millimetre range, many experimental setups show limitations. Position drifts on the order of hundreds of micrometres during thermal stabilization of the system can last for hours of expensive beam time. To compensate for drifts, sample tracking and feedback systems must be used. However, in complex sample environments where sample access is very limited, many existing solutions cannot be applied. In this work, we apply a robust computer vision algorithm to automatically track and readjust the sample position in the dozens of micrometres range. Our approach is applied in a complex sample environment, where the sample is in an ultra-high vacuum chamber, surrounded by cooled thermal shields to reach sample temperatures down to 2.5 K and in the center of a superconducting split coil. Our implementation allows sample-position tracking and adjustment in the vertical direction since this is the dimension where drifts occur during sample temperature change in our setup. The approach can be easily extended to 2D. The algorithm enables a factor of ten improvement in the overlap of a series of X-ray absorption spectra in a sample with a vertical size down to 70 µm. This solution can be used in a variety of experimental stations, where optical access is available and sample access by other means is reduced.

## Abstract 2

A computer vision algorithm for sample-position tracking on the order of dozens of micrometres is presented and its application to soft X-ray absorption spectroscopy in a high magnetic field, low-temperature endstation is demonstrated.

## 1. Introduction

X-ray spectroscopy techniques often have a similar setup consisting of a variable temperature insert (VTI) used to change the sample temperature in order to reach different phases of the compound under investigation (Dhesi *et al.*, 2010 ▸; Piamonteze *et al.*, 2012 ▸; Cezar *et al.*, 2013 ▸; Ohresser *et al.*, 2014 ▸; Barla *et al.*, 2016 ▸). Due to the contraction or expansion of the VTI (depending on which direction the temperature is varied), the sample position in relation to the X-ray beam will change. If stability in the sub-millimetre range is needed, many X-ray spectroscopy endstations do not have a reliable method for automatic sample realignment to facilitate this.

Many interesting compounds have an area size below 1 mm^2^. Some examples are thin layers from 2D van der Waals materials fabricated through exfoliation (Gish *et al.*, 2024 ▸; Cenker *et al.*, 2023 ▸), spin–orbit torque devices (Sala & Gambardella, 2022 ▸; Nasr *et al.*, 2023 ▸), certain single crystals (Klein *et al.*, 2021 ▸), among others. Inhomogeneous samples, where one is interested in investigating only particular areas, also become difficult if the sample positioning is not kept stable during measurements. Therefore, automatic sample-tracking and positioning systems are essential in order to make demanding experiments feasible within the beam time.

The element-specificity of X-ray magnetic circular dichroism (XMCD) or X-ray linear dichroism can provide valuable information to the systems mentioned above, but their lateral sizes are just at the limit of what can be measured in a reasonable amount of time in many XMCD endstations. In dichroic techniques, one measures a sequence of spectra with some external parameter changing between each spectrum. The parameter changed can be the magnetic field applied to the sample or the polarization of X-rays incident on the sample. The difference between these spectra measured under different conditions is the *dichroic signal*, which will, for example, allow us to quantify the magnetic moment of that sample. If sample drifts occur to the extent that the amount of sample illuminated by the X-ray spot size changes significantly, then consecutive X-ray absorption spectroscopy (XAS) spectra will have a different shape since they probe different amounts of a small sample or different parts of an inhomogeneous sample. When taking the difference between consecutive spectra, the dichroic signal will be masked by this undesired change in spectrum coming from position drift. Therefore, the smaller or the more inhomogeneous the sample, the more difficult it is to measure a reliable dichroic signal.

In this work, we have solved the problem of sample-position stability for samples with vertical sizes in the dozens of micrometres range. This solution was implemented in the X-Treme endstation (Piamonteze *et al.*, 2012 ▸). The sample is located inside an ultra-high-vacuum (UHV) chamber, surrounded by a superconducting magnet coil. The sample is attached to a VTI which allows access to sample temperatures down to 2.5 K. Using the approach presented here, we have decreased the changes in a series of XAS spectra caused by sample position drift by a factor of ten, measured in a sample with vertical size down to 70 µm.

This improvement is achieved thanks to automatic measurement and realignment of the sample position enabled by a computer vision algorithm which tracks the position of a marker mounted close to the sample investigated by X-rays. Implementing the position tracking into the data acquisition system leads to a highly accurate sample-position feedback loop. The precision in sample-position tracking is limited by the pixel size at the object plane. Tamtögl *et al.* (2016 ▸) used a computer vision algorithm for automatic sample alignment inside a UHV chamber. However, there the aim was not high position accuracy. Our approach aims to reach sample-position stability on the order of dozens of micrometres, which matches with other experimental factors of our system such as the X-ray beam spot size and positioning accuracy. However, the fundamental limits of the algorithm are dependent only on the image pixel size at the object position, as discussed in Section 5.

The paper is organized as follows: Section 2 provides the experimental details, Section 3 details the design and implementation of the computer vision algorithms (which we named *distect*), Section 4.1 evaluates the computer vision algorithms of *distect* in tracking the position of the sample holder inside the UHV environment by moving the VTI by known amounts and Section 4.2 details the results of a proof-of-concept experiment where measured XAS spectra in an artificially designed sample were acquired under real experimental conditions.

## 2. Experimental

### 2.1. Experimental chamber description

**Figure 1:**
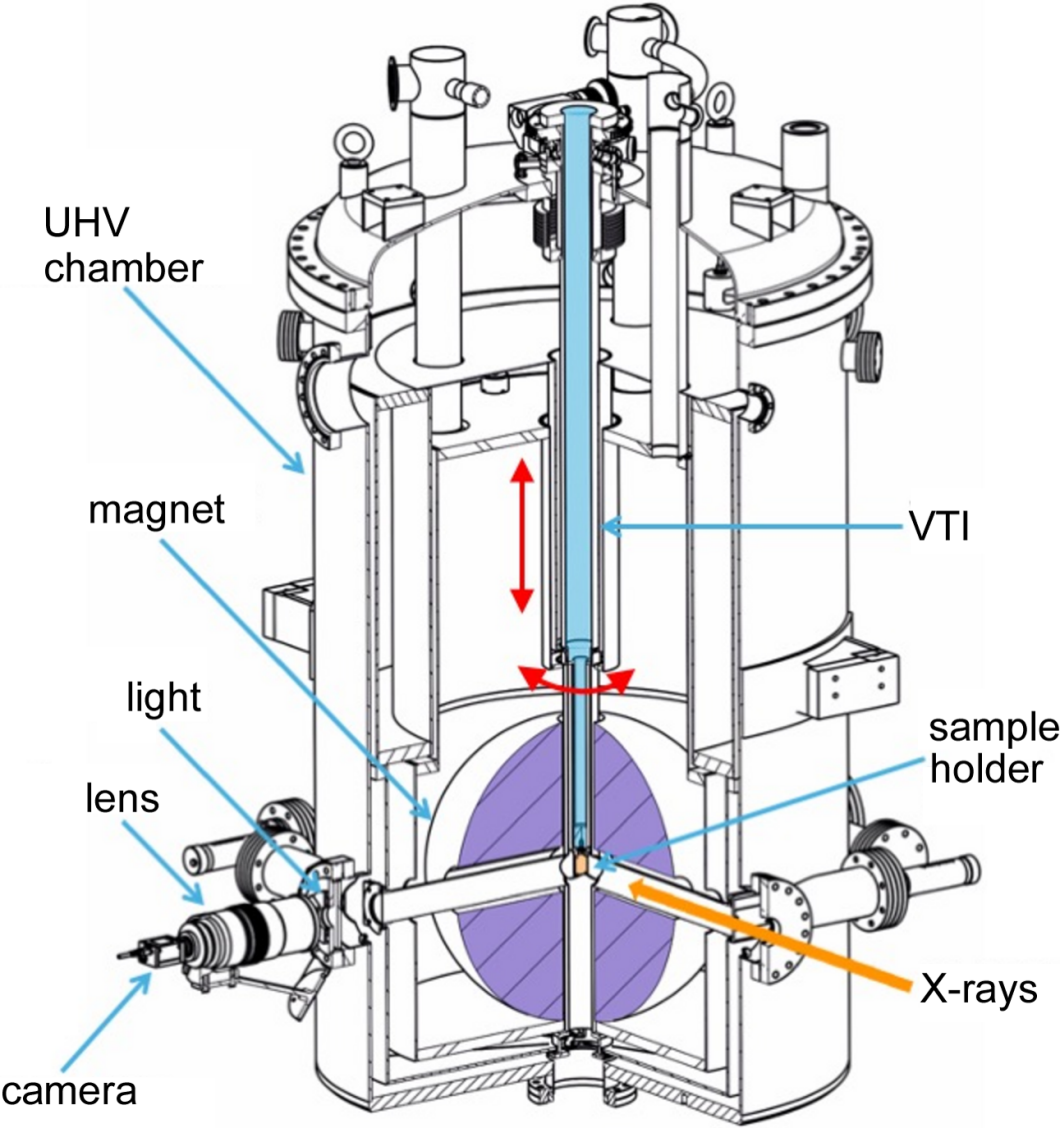
Side view with a cross-sectional cut of the endstation. The VTI is shown in light blue and the sample holder in orange. The red arrows show two motorized movements for the VTI and sample adjustment: vertical translation and rotation around the vertical axis. The camera, lens and ring light mounted on the side port and used by *distect* are also indicated. They are mounted at 90° to the incident X-ray direction shown by the orange arrow.

The measurements shown here were carried out in the superconducting magnet endstation installed at the X-Treme beamline (Piamonteze *et al.*, 2012 ▸) in the Swiss Light Source (SLS), Paul Scherrer Institute (PSI). A cross-sectional view of the endstation is shown in Fig. 1 ▸.

As shown in Fig. 1 ▸, the sample holder is attached by a screw connection to the bottom of the VTI. The sample position can be adjusted in relation to the fixed X-ray beam by three motorized movements. Two of these movements involve adjustment of the VTI and are indicated by red arrows in Fig. 1 ▸. Those movements are the vertical translation and the rotation around the vertical axis. The third sample adjustment is not indicated in the drawing; it involves moving the whole endstation horizontally in the direction perpendicular to the X-ray beam propagation.

The sample cooling works in the following manner. The VTI consists of two spaces (commonly called pots) separated by a needle valve. The top space is connected to a siphon where liquid helium is brought from the magnet bath to the VTI. This space is called the 4 K-pot. The 4 K-pot will have a flow of liquid helium close to atmospheric pressure every time the sample is cooled. The bottom part, below the needle valve, is called the 1 K-pot. When the lowest possible temperature at the sample is needed, the 1 K-pot is filled with liquid helium, the needle valve is closed and the 1 K-pot is pumped down with a roughing pump. The pumping allows the lower part of the VTI to be cooled below 4.15 K which is the temperature of liquid helium at atmospheric pressure. The temperature sensor at the 1 K-pot measures temperatures down to 1.4 K. Below the 1 K-pot there is a copper block thermally connected but electrically insulated from the 1 K-pot through a sapphire disk. The sample holder is attached to this copper block. The sample temperature, in this so-called base temperature condition, is 2.5 K. For temperatures above the base temperature the needle valve opening is changed in order to vary the amount of liquid helium that flows through the 1 K-pot. The temperature is then controlled by a heater located at the 1 K-pot using a proportional–integral–derivative loop control. The VTI from the X-Treme endstation can reach temperatures up to 360 K.

Changes in the sample vertical position in relation to the fixed X-ray beam occur when the sample temperature changes, either during cooling or warming cycles. This happens due to the contraction or expansion of the VTI length. The sample vertical position will change by up to 3 mm over the course of 40 min which is the time taken for cooling directly from 300 K to 2.5 K. After the sample temperature is stable, the sample vertical position is re-aligned in relation to the X-rays by a standard procedure. In our endstation, the alignment procedure is carried out by measuring and optimizing the sample XAS as a function of the vertical position. Visual adjustment using a camera can also be done. These alignment procedures work very well for samples with a vertical size above 1000 µm.

By design, the VTI is not a good thermal conductor since the room temperature at the top should not easily conduct to 1.4 K at the 1 K-pot. As a consequence, even after the sample temperature at the bottom is stable, the whole VTI length might still take hours to achieve a stable gradient temperature. Until this stable condition is achieved, there will be a drift in the vertical sample position on the order of hundreds of micrometres. This is not a problem if the sample measured is homogeneous and larger than 1000 µm, but is a problem for smaller or hetereogenous samples.

When using a focused X-ray beam on the order of dozens or hundreds of micrometres and if samples are smaller than 1000 µm in the vertical direction, the micrometres-drift described above causes the amount of sample illuminated by X-rays to change with time. *This is the drift we aim to compensate in this work.* The micrometres-drift depends more on the VTI temperature change history then on the actual sample temperature itself. The drift due to sample temperature change causes mostly changes in the sample vertical position. Therefore, the approach used here aims to measure and adjust the vertical sample position only. An extension to the 2D approach is discussed in Section 5.

### 2.2. Marker design

**Figure 2:**
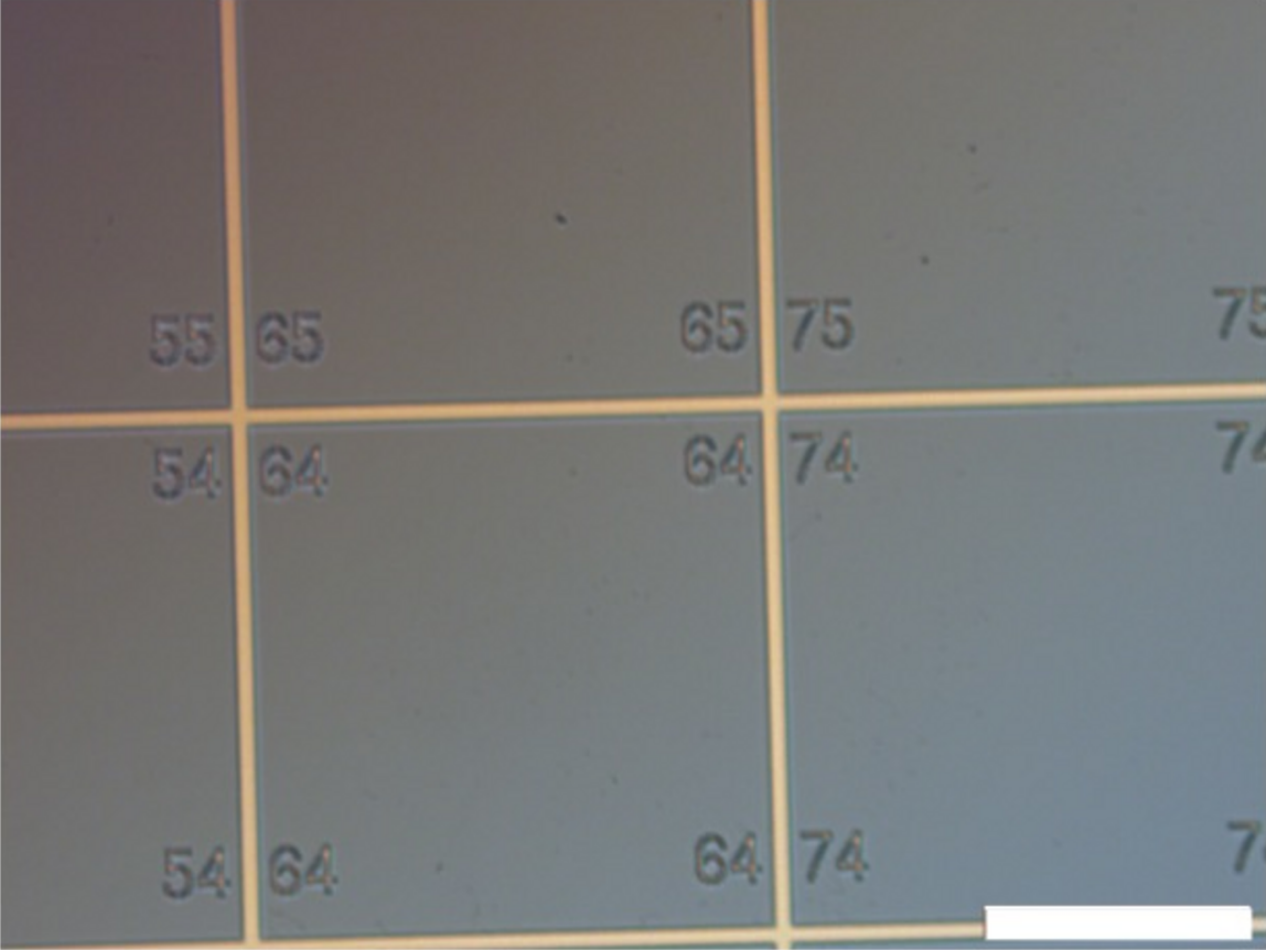
Photograph of the detail of one marker with Au lines deposited on Si forming a grid pattern. The lines are 35 µm wide with 1000 µm spacings. The scale is given by the horizontal white bar which is 500 µm.

The design chosen for the marker used to track the sample holder position is shown in Fig. 2 ▸. It consists of 35 µm-wide lines with 1000 µm spacings between them deposited on an Si wafer, forming a grid pattern. From here on, we will use the word grid when referring to the marker.

The grids were fabricated in PSI by electron beam lithography (EBL). We made two types of grids where the lines were made of Au (with thin Cr layers below and above) or only Cr. No difference in the *distect* performance between the two was noted. The fabrication steps are: a single polished 10 mm × 10 mm × 0.5 mm Si 〈100〉 wafer is covered with Cr 5 nm/Au 60 nm/Cr 2 nm or Cr 50 nm. Then the wafers are spin-coated with a negative electron-beam resist and exposed to the EBL tool to write the grid pattern. The surface is then developed and the parts not exposed in the electron beam are etched. An over-etching step is applied to Si to increase the contrast.

### 2.3. Optical setup

The optical access to the sample holder where the grid and sample are mounted is through a side viewport at 90° to the incoming X-ray beam, as shown in Fig. 1 ▸.

In our implementation, images of the grid are used to track the sample position. For imaging the grid two different optical setups were used for the results presented here. Both setups are similar and the software works equally well on both. The particularities of each setup are described in sequence. In both cases the distance from the grid to the camera sensor was approximately 700 mm. The pixel size in the object plane was calculated using the known distance of 1000 µm between the grid lines.

#### 2.3.1. Optical setup 1

The camera used was an Axis M1125 with RGB CMOS 1/3′′ sensor and a 2.6 µm pixel size. The camera was coupled to a Sigma zoom lens 70–300 mm f/4–5.6 DG macro (model 5A9080101). The images were taken with the lens set at 300 mm zoom and maximum aperture. This setting translated to a pixel size at the object plane of 6.5 µm. An achromatic doublet lens (Newport, PAC091AR.14) was mounted between the lens and the endstation window. A 5 mm-thick spacer ring was used between the lens and camera. Both the doublet and the spacer ring were necessary to reach focus at the grid position. The homogeneous illumination of the grid was achieved by an LED string fixed to the viewport where the camera is mounted (Fig. 1 ▸).

#### 2.3.2. Optical setup 2

The lens and camera were changed compared with optical setup 1 for convenience of operation in an experiment using X-rays. A Basler ace acA5472-5gm camera was used with a monocolor CMOS sensor of total size 13.1 mm × 8.8 mm and pixel size 2.4 µm × 2.4 µm. The camera was attached to a zoom lens 18–300 mm AFS Nikon AF-S Nikkor 18-300 mm f/3.5–6.3G ED DX VR. For this setup the pixel size at the object position was 10.9 µm with the lens at 300 mm zoom setting and with a fully open aperture. The grid illumination was carried out with a custom-made ring light to fit in the available window with an inner opening large enough for the camera view.

### 2.4. Design and fabrication of the sample for XAS measurements

**Figure 3:**
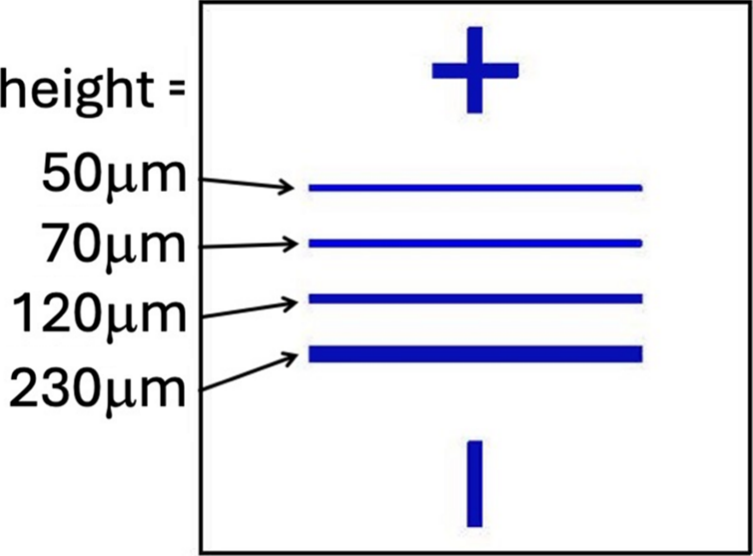
Sketch of the sample measured by X-rays. The blue lines were made of 30 nm-thick Cr. The cross on top and the vertical line at the bottom were used as guides during the manual alignment. The horizontal lines of variable heights were used as samples for XAS measurements. The line heights vary as indicated in the figure. Details on sample fabrication are given in the text.

We have fabricated a sample to be measured by X-rays as shown in Fig. 3 ▸. The blue lines are made of Cr. The sample was prepared by a lift-off process at the PSI clean room. A 10 mm × 10 mm × 0.5 mm Si 〈100〉 single-side polished wafer was spin coated with approximately 1.4 µm of positive resist. The resist is exposed using a laser direct writer tool and the desired pattern with the lines is written and developed. 30 nm-thick Cr was deposited on top. Then the lift-off process takes place using acetone in an ultrasonic bath. The lift-off removed the resist that was not exposed, leaving only the Cr lines.

### 2.5. Measurement geometry

In our setup, we can vary the incidence angle between the sample surface and the X-ray incident direction using the VTI rotation around the vertical axis, as shown by a red arrow in Fig. 1 ▸. The incidence angle is changed depending on the geometry required for each experiment. The most used incidence angles are either normal incidence, where the X-rays impinge at 90° with the sample surface, or grazing incidence, where the X-ray incidence is at 60–70° with the sample surface. Depending on the incidence angle, the sample holder surface facing the optical access viewport shown in Fig. 1 ▸ will be different. This requires the grid to be mounted differently, either on the same surface as the sample or on the side surface. Two different geometries were used in the results presented here.

#### 2.5.1. Geometry 1

**Figure 4:**
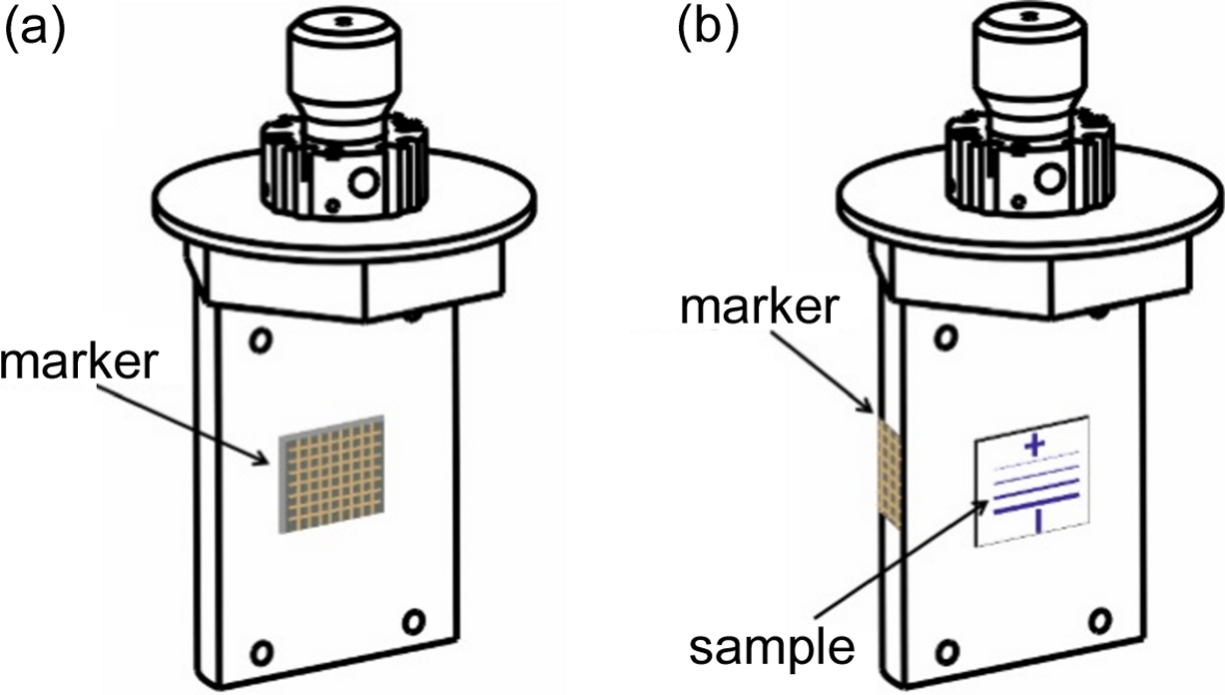
Sketch of two different sample holder mountings used here: (*a*) geometry 1 and (*b*) geometry 2. Details of both geometries are given in the text. (*a*) Mounting where only the marker was used for measurements without X-rays. (*b*) Mounting where both the marker and the sample measured by XAS are mounted.

The grid was mounted on the sample holder as shown in Fig. 4 ▸(*a*). The VTI angle was adjusted such that the grid was facing the camera mounted on the side port of the endstation. This type of geometry was used for off-line tests of *distect* (without X-rays), since in this case the sample holder surface is parallel to the incoming direction of the X-rays.

#### 2.5.2. Geometry 2

During XAS measurements, the grid and sample were mounted as shown in Fig. 4 ▸(*b*). This mounting geometry allowed the Cr sample to face the X-rays close to the normal, and the grid was facing the camera.

For measurements in grazing incidence, one could mount the grid on the same holder surface where the X-ray sample is mounted or in the back surface of the sample holder.

### 2.6. XAS measurement conditions

The XAS measurements were performed at the X-Treme beamline endstation of the Swiss Light Source. The beamline has the options of using a focused or defocused X-ray beam, as described by Piamonteze *et al.* (2012 ▸). Defocused X-rays are used to reduce radiation damage or charging and the typical sizes are 500 µm × 500 µm. Here we used the focused X-ray beam with 10 µm in the vertical and 250 µm in the horizontal.

The XAS spectra were acquired through total electron yield (TEY) signal and normalized by the incident X-ray intensity measured on an Au grid. The XAS spectra were acquired using on-the-fly mode (Krempaský *et al.*, 2009 ▸). Each single spectrum took 3 min. The sample temperature was 300 K throughout the measurements. The sample temperature was 180 K before the measurements, therefore the VTI was still reaching thermal equilibrium during the XAS measurements.

### 2.7. Software implementation

The computer vision algorithms for sample tracking – also called dislocation detection (*distect*) – have been implemented in Python 3 using OpenCV (Bradski, 2000 ▸). The Python program can be executed as a standalone program but can be imported as a Python module as well, which facilitates the integration with our *EPICS* (EPICS, 2024 ▸) based control environment. The control program is written as a *PCASpy* (PCASPY, 2024 ▸) server, which feeds the image data to the program and makes the parameters and results accessible by any *EPICS* client. The output from *distect* which provides the position of the grid at a given moment in relation to a reference image was accessed as an *EPICS* channel. The reference image was taken each time the software was reset. The reset function was also set as an *EPICS* channel. The reset was used when large changes on the VTI vertical position are made, for example to access different samples mounted on the sample holder.

## 3. Computer vision algorithms for sample tracking

The basic idea of the algorithm is to automatically track the movement of markers mounted on the sample holder as the sample position changes over time. For simplicity as well as robustness, in our approach, the marker looks like the grid shown in Fig. 2 ▸. Hence, we can phrase it as a computer vision algorithm, which basically consists of line-grid detection and associating intersection points of the grid over time to track the displacement of the probe and act accordingly to compensate for it. In other words, our approach does not require a complex object detection algorithm but a high-precision line detection algorithm that is robust against impurities in the image. Hence, in this work we are using a modern data science approach (Braschler *et al.*, 2019 ▸) to tackle a real-world problem.

In order to detect the line positions of the grid, a series of algorithmic steps are carried out in the image. These steps are explained in the following paragraphs.

### 3.1. Image pre-processing

Images can carry a variety of noisy influences that impede the performance of computer vision algorithms. One such issue can be missing contrast between the lines and the Si surface. In order to improve the contrast, we performed image intensity re-scaling by scaling the detected contrast values to the full range between the minimum and maximum value. To counter possible physical imperfections on the marker, we applied a 3 × 3 median filter to the image. It has the effect of smoothing the image. Thin lines such as scratches and small dots such as dust particles are fused together with their surrounding, making them less prominent.

### 3.2. Edge extraction

**Figure 5:**
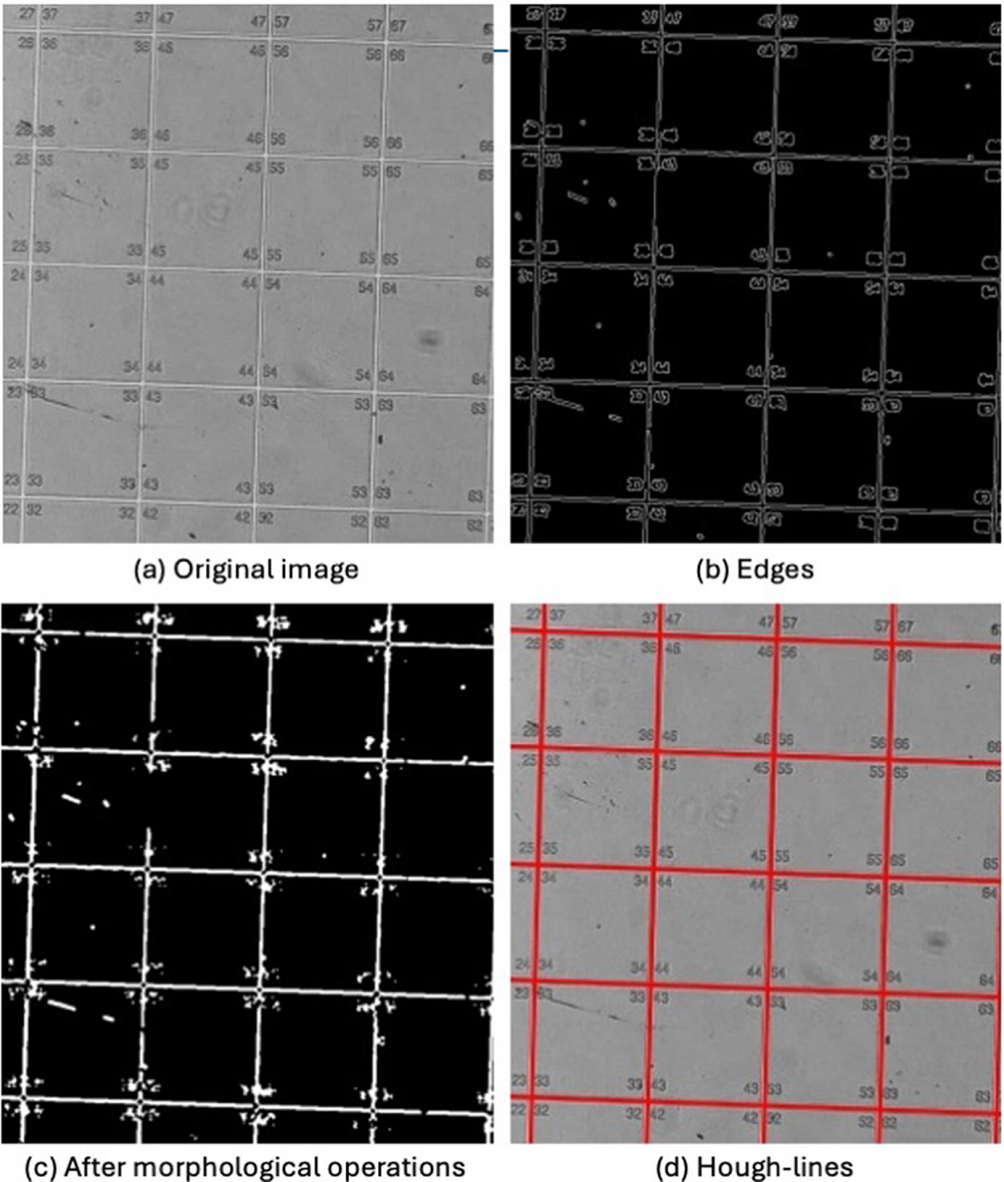
End-to-end pipeline of the computer vision algorithm to detect grid lines.

The next step is to extract the edges of the image using the *Canny* algorithm (Canny, 1986 ▸). The edge image is a reduction of the original image to emphasize high differences in the gray values of neighboring pixels, *i.e.* edges of objects [see Fig. 5 ▸(*b*)]. Homogeneous regions of the image are removed. A binary image is obtained after thresholding. In order to close small gaps and remove clutter, morphological operations are used and the results are shown in Fig. 5 ▸(*c*).

### 3.3. Line detection

The next step is to extract the grid lines using the *Hough Transform* (Duda & Hart, 1972 ▸). Each edge pixel from the binary edge image votes for all possible lines this pixel might be part of. For straight lines this parameter space is defined by the distance (*d*) and angle (θ). Hence, one edge pixel is transformed into a sinusoidal curve in a parameter space. An accumulator array is used to record where these curves intersect, with the highest intersections indicating the most likely lines. The peaks in this array correspond to the detected lines in the original image, making the *Hough Transform* effective at identifying lines even in noisy or cluttered images. The lines found for the example image are shown overlapped with the original image in Fig. 5 ▸(*d*); note that the lines do not need to be perfectly horizontal/vertical in relation to the sensor frame to be detected.

### 3.4. Intersection calculation

The next step is to calculate the grid intersection points and the conversion factor from pixels to millimetres. The intersections are needed to measure the dislocation between two images.

For the intersection calculation, the straight line parameters of an image are grouped horizontally and vertically by the angle. Then the intersection points are calculated by stepping through each of the vertical lines and calculating its intersections with all horizontal lines.

### 3.5. Dislocation calculation

**Figure 6:**
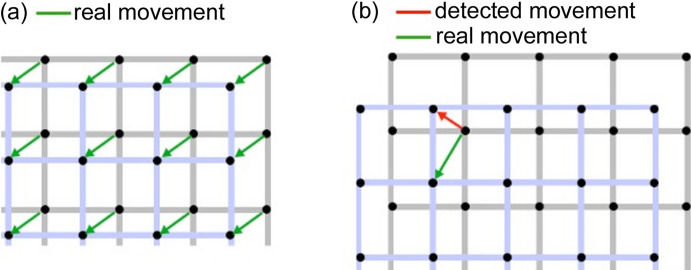
Scheme showing dislocation detection between two images. The grid in the reference image is shown in gray and the grid from the new image is represented by blue lines. Green arrows indicate the correct intersection correspondence between the two images. In (*a*)/(*b*) the movement is smaller/larger than half the distance between the lines. The red arrow in (*b*) indicates the software-calculated distance, which does not correspond to the real distance in this case.

After having transformed each image to a list of intersections, the total dislocation is calculated. Each intersection point of an image is compared with all intersection points in the following image and the distances between them are calculated. The shortest distance between two intersection points indicates the correspondence of the two intersections in the two images. This is schematically represented in Fig. 6 ▸(*a*). After having found all correspondences between the intersection points of two images, the dislocation values are calculated. The final dislocation is calculated as the median of all individual intersection dislocations for each image.

The maximum detectable dislocation value that can be obtained by *distect* is given by half the distance between the grid lines, which is 0.5 mm for the grid we used. This limit arises from the fact that, if the dislocation between two images is larger than 0.5 mm, the closest intersection found by the algorithm will not be the correct one. This situation is represented in Fig. 6 ▸(*b*) where the red arrow shows the distance the algorithm would calculate in this situation while the green arrow would be the correct distance. This does not pose a limitation for our application, since the movement between images will likely be smaller than 0.5 mm. As shown in Fig. 6 ▸, the software calculates both vertical and horizontal distances, but we currently only use the vertical distance. For the horizontal distance to be meaningful, one would need to have the view from the X-ray incoming direction, this option is discussed in Section 5.

## 4. Results and discussion

We have performed two experiments to check the performance and limits of the algorithm *distect* described in Section 3. In the first experiment, presented in Section 4.1, we checked the ability of the algorithm to correctly calculate the movements of the grid. This is done at the X-Treme experimental chamber but independent of X-ray measurements. In the second step, presented in Section 4.2, we use *distect* to measure unknown sample drifts under real experimental conditions in parallel with XAS measurements. In this experiment, we test the limits of our setup, which is defined not only by the performance of *distect* but also the precision of our sample alignment motor and the X-ray spot size.

### 4.1. Offline evaluation of the tracking algorithm

The aim of the measurements presented in this section is to evaluate how well *distect* works in calculating set movement between images where the amount of movement varies from dozens of pixels (hundreds of micrometres in the object plane) down to a single pixel (dozens of micrometres in the object plane).

#### 4.1.1. Results

The measurements were carried out offline, meaning without X-rays coming in the chamber. The images were taken at the X-Treme endstation (Fig. 1 ▸) using optical setup 1 and geometry 1 described in Section 2.

**Figure 7:**
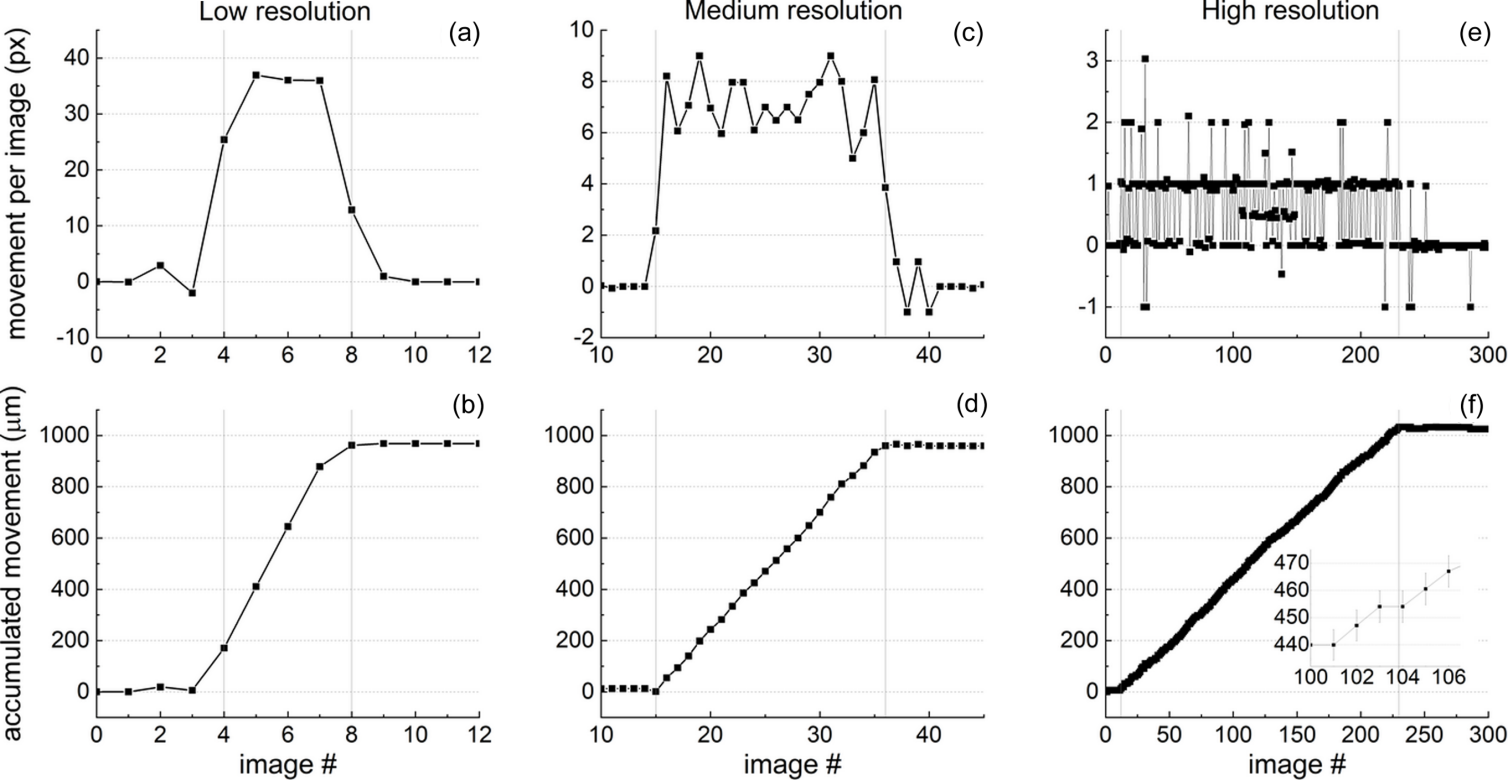
Movement calculated by *distect* between consecutive images plotted against the number of images. The movement in pixels calculated between each image and its predecessor is plotted on top: panels (*a*), (*c*), (*e*). The accumulated movement in micrometres is plotted at the bottom: panels (*b*), (*d*), (*f*). The conversion factor is 0.154 px µm^−1^. Gray vertical lines mark the range when the VTI was in movement. The average movement speed was (*a*)–(*d*) 200 µm s^−1^ and (*e*)–(*f*) 20 µm s^−1^. The image taking frequency was (*a*)–(*b*) 1 image s^−1^ and (*c*)–(*f*) 4.4 images s^−1^.

Three series of images were saved where the average step sizes of the VTI movement between each single image were approximately 200 µm, 48 µm and 4 µm. We call those low-, medium- and high-resolution movement series. The images were analyzed by *distect* in the order they were saved. The results of this analysis are plotted in Fig. 7 ▸. The range between the two vertical gray lines in each panel of Fig. 7 ▸ mark the images taken while the VTI was in movement. Figs. 7 ▸(*a*), 7 ▸(*c*) and 7 ▸(*e*) show the calculated difference in the grid vertical position between each image and its predecessor in image pixels. Figs. 7 ▸(*b*), 7 ▸(*d*) and 7 ▸(*f*) show the accumulated movements, which are the sums of the values from the panels above, respectively. In Figs. 7 ▸(*b*), 7 ▸(*d*) and 7 ▸(*f*), the output is converted to the actual distance in micrometres in the object plane.

For the data shown in Fig. 7 ▸(*a*) and 7 ▸(*b*), the average motor speed was 200 µm s^−1^ with an image taking frequency of 1 s^−1^. It can be seen in Fig. 7 ▸(*b*) that the calculated accumulated movement adds up in steps of around 200 µm as expected in these settings. Fig. 7 ▸(*a*) shows that there is a period of positive and negative acceleration at the beginning and end of the movement with a stable output in the center. The difference in the grid movement between each image during the stable VTI movement is around 36 px. Outside the VTI movement range, Fig. 7 ▸(*a*) shows a difference of 0 between the the grid images, as expected. The accumulated movement is underestimated by 3.4% in relation to the motor target movement of 1000 µm.

In Figs. 7 ▸(*c*) and 7 ▸(*d*) we show the medium-resolution series. The calculated movement between each individual image shown in Fig. 7 ▸(*c*) averages to 7 ± 1 px during the VTI movement. After the VTI stops, the variation in reading between each image is mostly around 0, with some points scattering to ±1 px. The accumulated movement calculated by *distect* is 3.1% below the motor target movement of 1000 µm.

Figs. 7 ▸(*e*) and 7 ▸(*f*) show the results for the high-resolution series, when the average movement between each image is below 1 px. It is surprising that even for this setting the prediction of movement by *distect* still works. The accumulated movement is 3.6% above the movement target of 1000 µm. Fig. 7 ▸(*e*) shows that around every other image the movement of 1 px is registered, those values then accumulate to close to the correct target movement. The error bars plotted in the inset correspond to the standard deviation of the median of all intersection points in each image, which is around 1 px.

#### 4.1.2. Discussion

For the three series the accumulated dislocation calculated by the software returns values close to the total of 1000 µm with over or underestimation around 3.1–3.6%. This corresponds to an error of 4.8–5.5 px or 31–36 µm in the object plane. This error is large compared with the error observed for each image calculation, which is around 1 px. Here it is difficult to disentangle if the disagreement comes from the step motor reproducibility or from *distect*. Overall the results are very good and encouraging. The approach used by *distect* has been able to predict the total motor movement even in the case when the difference between individual images was below 1 px. The noise in the *distect* output is around ±1 px.

### 4.2. Application to XAS measurements

In this section, we show how *distect* works in detecting and being used to readjust the sample vertical drifts, consequently improving the reproducibility between consecutive XAS spectra.

#### 4.2.1. Results

For the grid image acquisition, optical setup 2 described in Section 2 was used. The *distect* implementation combined with *EPICS* (as described in Section 2) was used. *distect* acquires live images of the grid, analyzing and calculating the vertical movement in relation to a reference image. There was a vertical position output about every second. In order to reduce reflections and improve contrast of the image, the grid was covered with ∼60 nm of Si_3_N_*x*_ as an anti-reflection cover. The Si_3_N_*x*_ thickness was calculated to reflect the main emission wavelength of 460 nm from the LED light used (Leverant *et al.*, 2023 ▸).

We performed XAS measurements of an artificially designed sample consisting of horizontal Cr lines of variable width as shown in Fig. 3 ▸. The sample fabrication details are described in Section 2. Each horizontal line served as an individual sample for the XAS measurements. Since the drift we aimed to compensate occurs in the vertical, we wanted to have comparable samples of different vertical lengths. This sample design allowed us to check the limit in the sample vertical length where our implementation shows significant improvements in the reproducibility of consecutive XAS spectra.

To reproduce real experimental conditions where drifts occur after a sample temperature change, the sample was warmed from 180 K to 300 K before XAS measurements. Once at 300 K, a 30 min waiting period was used to assure the sample temperature was stable. However, this is not long enough for the whole VTI to reach its stable temperature gradient, which takes several hours. Therefore, drifts of micrometres still occurred during the measurements.

The sample and grid mounting were as shown in Fig. 4 ▸(*b*) and described in geometry 2 in Section 2. As the grid was mounted on the side surface of the sample holder it had to be cut down to ∼3–4 mm (width) × 7 mm (height). There must be a minimum of two horizontal lines in the camera field of view for *distect* to be able to measure the vertical movement. Since the calculated distance is the median of all intersection point differences between two images (as explained in Section 3), the number of intersection points should be as large as possible.

**Figure 8:**
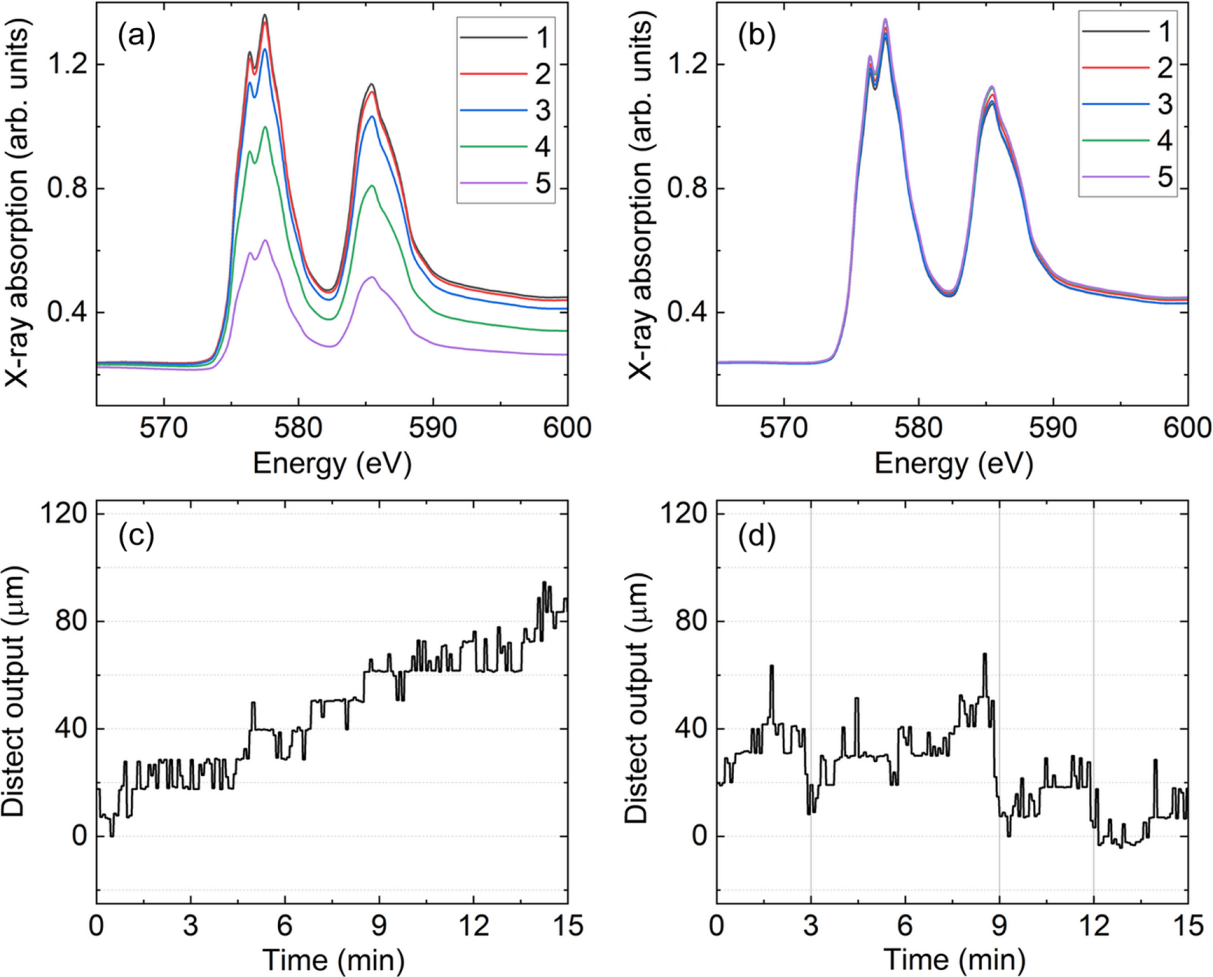
(*a*)–(*b*) XAS at the Cr *L*_3,2_-edges for a sample of 120 µm height. The legend indicates the sequence of measurement. (*a*) Shows results without automatic adjustment and (*b*) shows the results with automatic adjustment. (*c*)–(*d*) Position measured by *distect* as a function of time during the measurements shown in (*a*)–(*b*), respectively. In *(d*) the vertical gray lines show when the vertical sample position was adjusted.

The Cr XAS spectra measured for lines of 120 µm, 70 µm and 50 µm in the vertical are presented in Figs. 8 ▸(*a*)–8 ▸(*b*), 9 ▸(*a*)–9 ▸(*b*) and 10 ▸(*a*)–10 ▸(*b*), respectively. Taking for example Fig. 8 ▸(*a*), it can be seen that the Cr spectrum consists of two peaks coming from the Cr *L*_3_-edge around 577 eV and the Cr *L*_2_-edge around 585 eV. These peaks in X-ray absorption correspond to resonant electronic transitions from the Cr 2*p* core level to the unoccupied Cr 3*d* states. The fine structure at the Cr *L*_3_-edge is likely to come from a Cr oxide layer forming at the surface (Bastidas *et al.*, 1998 ▸; Aksoy *et al.*, 2010 ▸).

Let us consider in detail the results presented in Fig. 8 ▸ for the 120 µm line. Figs. 8 ▸(*a*) and 8 ▸(*b*) both show a series of five spectra taken in sequence. In Fig. 8 ▸(*a*), the sample position was aligned once before the beginning of the XAS measurements series. In Fig. 8 ▸(*a*) the XAS spectra intensity is continuously decreasing. This happens due to the drift in the sample vertical position caused by the VTI reaching thermal stability. In Fig. 8 ▸(*b*), after the initial alignment, the output of *distect* was used to keep the sample aligned automatically. Comparison between Figs. 8 ▸(*a*) and 8 ▸(*b*) show that there is an improvement in the overlap of the raw XAS spectra, when the sample-position tracking provided by *distect* was used to automatically keep the sample aligned.

Figs. 8 ▸(*c*) and 8 ▸(*d*) show the output of *distect*, acquired in parallel to the XAS measurements shown in Figs. 8 ▸(*a*) and 8 ▸(*b*), respectively. Automatic alignment during the measurements shown in Fig. 8 ▸(*b*) was carried out in the following way. At the end of each individual spectrum the output of *distect*, shown in Fig. 8 ▸(*d*), was read. If the movement measured by *distect* was larger than 22 µm (which corresponds to 2 px at the object plane), the sample vertical position was adjusted by the value measured by *distect*. Therefore, there was an opportunity to adjust the sample vertical position every 3 min, which is the time taken to collect a single XAS spectrum. The points at which the sample vertical position was adjusted are shown in Fig. 8 ▸(*d*) by gray vertical lines.

For the spectra plotted in Fig. 8 ▸(*a*) we observe a relative decrease in the Cr *L*_3_-edge jump which is up to 55% by the end of the scan series. Edge jump is the difference in intensity between the pre-edge region around 570 eV and the peak of XAS spectra around 578 eV. In Fig. 8 ▸(*b*), where the sample position was automatically adjusted, the largest relative change at the Cr *L*_3_-edge is 6%.

**Figure 9:**
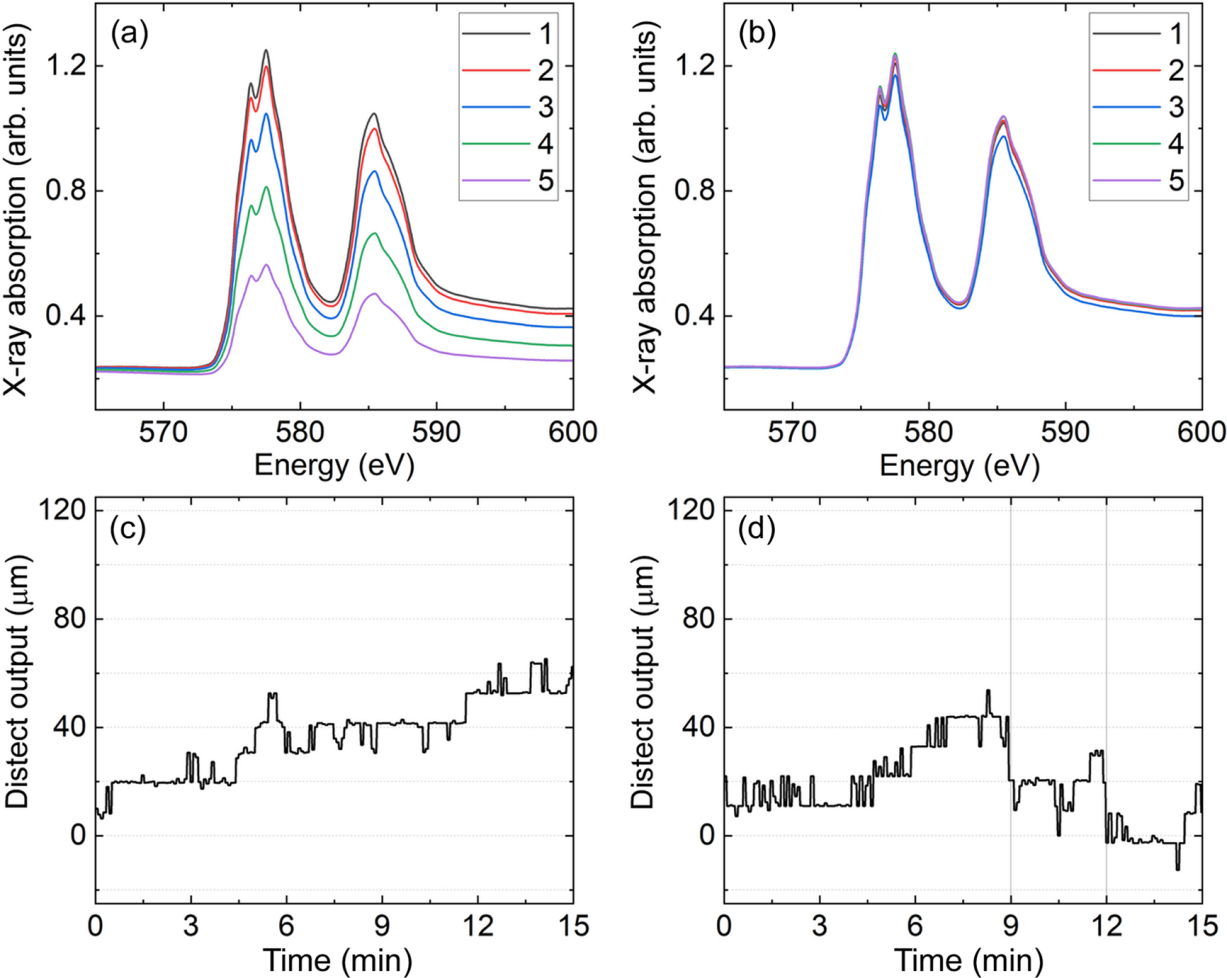
(*a*)–(*b*) XAS at the Cr *L*_3, 2_-edges for a sample of 70 µm height. The legend indicates the sequence of measurement. (*a*) Shows results without automatic adjustment and (*b*) shows the results with automatic adjustment. (*c*)–(*d*) Position measured by *distect* as a function of time during the measurements shown in (*a*)–(*b*), respectively. In (*d*) the vertical gray lines show when the vertical sample position was adjusted.

Similar changes are observed in Fig. 9 ▸ where the spectra for the 70 µm Cr line are shown. In Fig. 9 ▸(*a*), the fifth spectrum has a reduction of the *L*_3_-edge jump which is 55% of the first. In Fig. 9 ▸(*b*), the largest difference in the *L*_3_-edge jump is found between the third and first spectrum. The *L*_3_-edge jump of the third spectrum is 5% smaller than in the first one. Indeed, the data plotted in Fig. 9 ▸(*d*) show that the largest relative movement measured by *distect* is between 6 min and 9 min, when the third spectrum was measured. For the remaining spectra shown in Fig. 9 ▸(*b*), the relative difference to the first spectrum is at most 3% at the Cr *L*_3_-edge.

**Figure 10:**
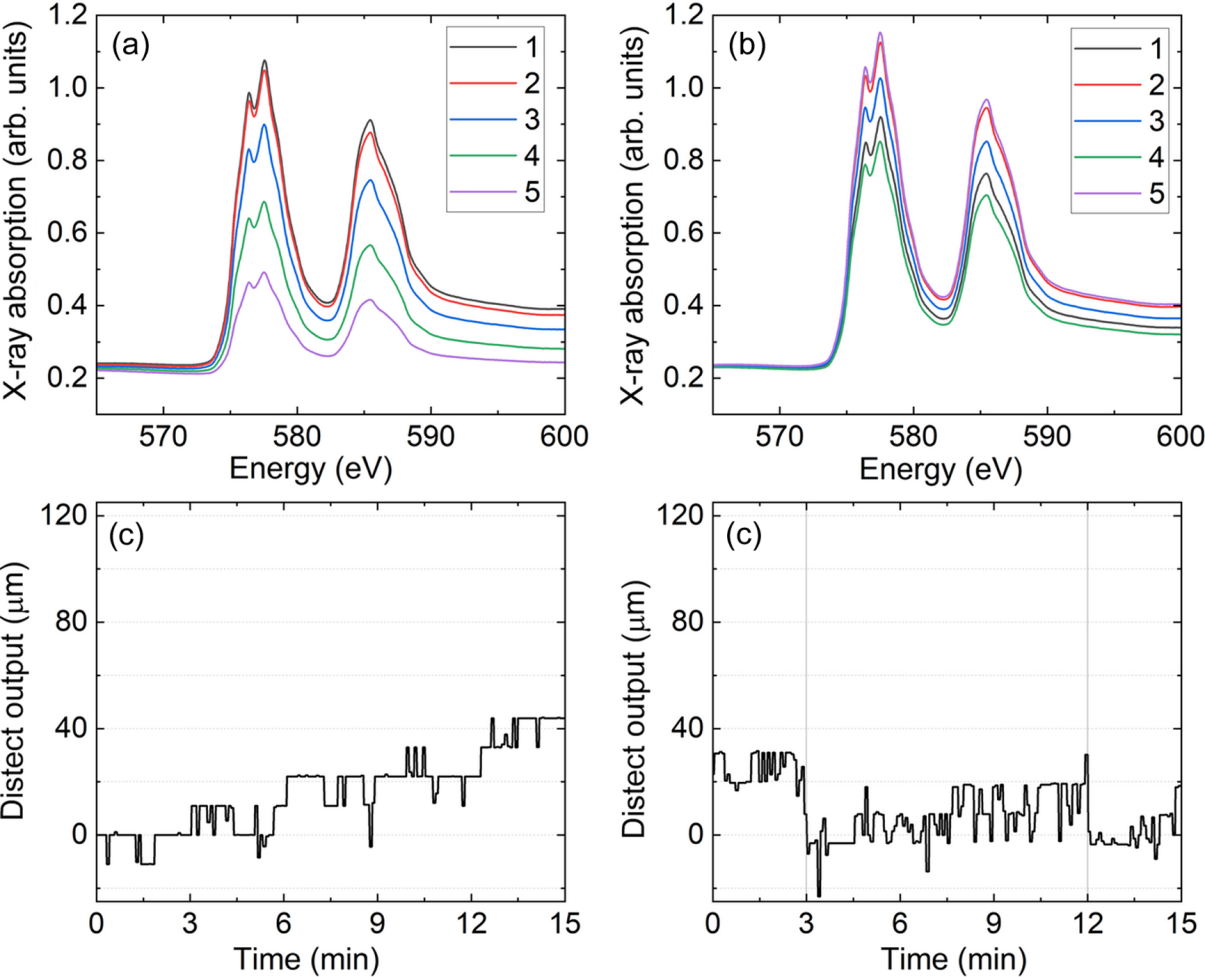
(*a*)–(*b*) XAS at Cr *L*_3, 2_-edges for a sample of 50 µm height. The legend indicates the sequence of measurement. (*a*) Shows results without automatic adjustment and (*b*) shows the results with automatic adjustment. (*c*)–(*d*) Position measured by *distect* as a function of time during the measurements shown in (*a*)–(*b*), respectively. In (*d*) the vertical gray lines show when the vertical sample position was adjusted.

The XAS spectra measured for the Cr line with 50 µm height is shown in Fig. 10 ▸. The adjustment using *distect* also helped to reduce the changes in the *L*_3_-edge jump from 50% in Fig. 10 ▸(*a*) to 25% in Fig. 10 ▸(*b*), when comparing the first and fifth spectra. However, the changes between consecutive spectra is clearly still too high even with the automatic alignment in Fig. 10 ▸(*b*). This result is expected since we have set the movement every 22 µm which is close to half the vertical length of the sample.

#### 4.2.2. Discussion

Figs. 8 ▸(*a*), 9 ▸(*a*) and 10 ▸(*a*) show a typical example of the problem we would like to minimize in this work. If nothing has been actively changed in the experimental conditions between each spectrum, then all spectra should overlap with each other. However, Figs. 8 ▸(*a*), 9 ▸(*a*) and 10 ▸(*a*) show this is clearly not the case. Figs. 8 ▸(*c*), 9 ▸(*c*) and 10 ▸(*c*) hint to what is happening. The grid position measured by *distect* has moved by 90 µm, 60 µm and 45 µm during the series of five spectra measured in Figs. 8 ▸(*a*), 9 ▸(*a*) and 10 ▸(*a*), respectively. Since the sample is moving and as time passes, the X-rays illuminate less and less of the Cr line. The total movement for each series is different because the drift will change with time. The closer in time the measurement is done to the temperature change, the faster the drift will be. The amount of drift shown in Figs. 8 ▸(*c*), 9 ▸(*c*) and 10 ▸(*c*) does not pose a problem for samples larger than 1000 µm in the vertical. However, it makes the measurements of samples on the order of dozens of micrometres in the vertical challenging, as illustrated by the data in Figs. 8 ▸(*a*)–10 ▸(*a*).

If effects like those shown in Figs. 8 ▸(*a*), 9 ▸(*a*) and 10 ▸(*a*) occur during a dichroic measurement, there will be a large difference between the spectra coming from sample movement. This unwanted difference signal will mask the dichroic signal the user intends to measure. Therefore, the much better overlap observed in Figs. 8 ▸(*b*) and 9 ▸(*b*) will allow dichroic measurements to be performed in future experiments for samples as small as 70 µm in the vertical direction. The measurements in Figs. 8 ▸ and 9 ▸ show that, for samples with vertical sizes of 120–70 µm, the XAS spectra overlap is 10× better when *distect* is employed to automatically align the sample and compensate the drifts before each single spectrum.

We have shown XAS measurements carried out in an artificially designed sample. This was done to verify the lower limit in the vertical sample dimension where our approach works. The sample-position measurements developed here can be applied to samples of arbitrary shapes. The only precondition for *distect* is to place the marker with a grid pattern on the same sample holder as the investigated sample and within the field of view of the camera.

The approach presented here is also beneficial for the measurements of large samples. The automatic alignment provided by *distect* will save time of manual realignment each time the sample temperature changes. Moreover, XAS measurements during a controlled temperature ramp can be performed in a wide temperature range without the need of interruption for realignment.

## 5. Conclusions

In this work, we demonstrated how to improve the sample drift measurement and automatic alignment in our endstation. An improvement in spectra overlap by a factor of ten is observed for samples down to 70 µm in the vertical. As shown in Section 4.1, the noise in the movement detection of *distect* is around 1 px. Therefore, a possible improvement of our setup would be to increase image magnification, which means decreasing the pixel size at the object position. This can be achieved with microscopic lenses. In our case, where the sample is relatively far from the optical access window, we could use an ultra-long-distance zoom microscope. The sample length of 70 µm corresponds to around 6.4 px in the object plane. This limit is given by a convolution of our X-ray spot size and the VTI motor reproducibility together with the smallest movement we could detect with *distect*. Therefore, an improvement in the magnification optics only makes sense if the X-ray spot size and movement reproducibility match the pixel size in the object plane.

The focus of our approach was on tracking and adjusting the sample vertical position since this is the dimension where most of the drift occurs in our setup. If needed, the approach can be easily extended to a 2D adjustment. The grid design of the marker allows for measuring movement also in the horizontal. *distect* already calculates distances in the horizontal. To implement a horizontal adjustment, the camera needs to have a view from the X-ray beam direction. To achieve this, one could introduce a mirror upstream from the sample at the X-ray beam path, with an opening to allow the X-ray beam to go through. The mirror would be mounted at 45° with the X-ray incidence direction reflecting the sample front image to a viewport and camera mounted at 90° with the X-rays.

Another improvement which can be implemented would be to measure each energy spectrum in less time (here we used 3 min for each scan). This allows the adjustment to be done in shorter intervals. This is easy to implement as we are not at our monochromator speed limit. Continuous alignment of the sample in closed loop mode is, in principle, possible. It would be necessary to test if the movement during measurements causes noise in the TEY signal. Moreover, it is likely to be beneficial to average some of the outputs from *distect* to decrease the noise and have more accurate output on the sample position.

One limitation of our implementation is that visible light is required inside the experimental chamber to illuminate the grid. This can pose a problem if the XAS spectra detection is done by photo-sensitive detectors. These detectors would register a large background signal coming from the visible light. Photodetectors are used, for example, in the case of XAS measured by X-ray fluorescence or transmission or in the case of X-ray scattering (elastic and inelastic) techniques. This limitation could be overcome if a filter could be mounted in front of the photodetector. The filter would need to be optimized to absorb the visible light but transmit the X-rays.

In summary, we have shown that the use of an inexpensive experimental setup with a robust implementation of computer vision algorithms can be used to successfully measure the sample position in real time in a non-invasive way. Our implementation was tested in a sample inside a low-temperature, high magnetic field, UHV chamber, where sample access is very limited. The use of *distect* will widen the type of samples that can be measured at the X-Treme beamline after the SLS upgrade. Our focus here was on dichroism measurements, but the need for counteracting sample-position movement caused by temperature change applies to many spectroscopy endstations. Therefore, the solution presented here will be useful for a wide variety of experimental setups where optical access is available.
